# The role of consciousness in triggering intellectual habits

**DOI:** 10.3389/fnhum.2014.00312

**Published:** 2014-05-21

**Authors:** Javier Sánchez-Cañizares

**Affiliations:** Mind-Brain Project, Institute for Culture and Society (ICS), University of NavarraPamplona, Spain

**Keywords:** consciousness and truth, intellectual habits, inhibitory control, post-error slowing, attentional orientation

Why does a theoretical physicist describe a system from the point of view of its mathematical symmetries instead of performing a numerical simulation? The researcher chooses the strategy that should provide the most relevant information. Nevertheless, the different strategies have not always been available. Scientific discovery has actually happened in history thanks to the creativity of a good number of thinkers, whose insights proved to be decisive for the developing of new branches of science. New ways of confronting well-posed problems may eventually become intellectual habits of generations of scientists, but such habits can only develop after checking the validity of these new perspectives. What the history of science shows is somehow reproduced in the learning process. “Knowing-about” forms the heart of standard education: students can learn and be tested on it. But success in examinations gives little indication of whether that knowledge can be employed when required, which is the essence of “knowing-to.” When must a specific intellectual habit be called upon? Knowing-to has to do with the conscious use of cognitive habits. It implies a conscious judgment of an upper-level truth about the problem. The practice of reflection is a means to help students improve their knowing-to act in the moment because the triggering situation for the enactment of a new behavioral schema must be conscious. Thus being explicit about one's own thinking improves mathematics teaching and learning (Lim and Selden, 2009). The “aha” moment is experienced by someone who learns a new strategy to tackle a problem, develops a new intellectual habit and knows when to use it. This paper comments on neuroscientific support of the “aha” moment through the inhibition mechanisms of the brain.

Inhibitory control is an executive process involved in attention, self-regulation, and consciousness. Intelligence is closely tied to the ability to inhibit a misleading behavior, judgment, or strategy, and inhibition is precisely the cognitive mechanism that should allow one to redirect attention toward logically relevant issues. It seems to be crucial in order to validate and activate a new mode of thinking. Houdé's group experimentally showed that the biased (spatial) to logical shift in the way of solving a logical problem with geometrical objects of different colors and shapes is a specific consequence of executive training in matching-bias inhibition. The relevant point is that inhibition allows subjects to redirect attention to the logically correct shapes, a shift process in which the activated brain networks can change radically in the same subjects depending on their ability to inhibit a misleading strategy (Houdé et al., 2000). Posterior-to-anterior reconfiguration of the activated brain regions brought about by inhibitory control might be the neural correlate of human abstraction, the ability to break away from perceptual biases during cognitive development. Houdé's training helps the subject to be conscious of the implicit mistake he or she is making and, therefore, what he or she must do to avoid the trap.

Recent empirical work clarifies the specificity of high order cognitive process enacting inhibition. Overall, how errors impact the processing of subsequent stimuli and in turn shape behavior remains unresolved. However, the literature documents two main mechanisms when correcting errors: bottom-up, automatic and top-down, controlled forms of inhibition (Spierer et al., 2013). Actually, many results are interpreted in terms of a shift from a fast automatic to a slow controlled form of inhibitory control induced by the detection of errors, which could have been caused by an attentional modulation (Manuel et al., 2012). Some experiments on post-error slowing in subjects support the view that outcome expectancy (not accuracy) is essential for such effect. Post-error slowing is caused by attentional orienting to unexpected events and not by a strategic adjustment of cognitive nature (Núñez Castellar et al., 2010). Then, there seems to be a qualitative criterion for the subject to decide when accuracy is important because expectancy is not fulfilled. In short, the subject has expectancy. He or she looks for an *adequacy* with the input signal. And he or she needs attentional reorientation when this adequacy is not satisfied.

Intentional inhibitory control exists whenever the subject's creativity and checking of the truth (adequacy) is required in a new problem. Deliberate inhibition is necessary even if the subject has the proper *knowledge about*, but does not *know when to* use it. In other words, inhibition is an effect of *detecting error and shifting to a new cognitive strategy* in which awareness is necessary for shifting from fast to slow forms of control. The role of consciousness is related to the inhibition of the common (habitual) cognitive strategy, which would allow for the activation and recruiting of the brain areas involved in a new type of reasoning. But the specific “judgment of truth” to accept or reject a new strategy turns out to be an exclusive feature of consciousness. This explains, for instance, why the validation of a mathematical generalization cannot initially be a habit. Theoretical scientists must beforehand *judge* the relevant strategy for tackling a problem and then make *conscious* use of an intellectual habit—which might be new in the case of new theoretical discoveries—to try to solve it. To sum up, human consciousness—as something different from a pure brain state—is required in order to establish the validity of a new theoretical perspective. Once this is reached, new habits may be at work. Consciousness mediates between the unconscious formation of new ideas and the development of new habits, which need check of the new ideas' adequacy in order to be prompted. Therefore, consciousness is the precursor for the activation of intellectual habits.

## Conflict of interest statement

The author declares that the research was conducted in the absence of any commercial or financial relationships that could be construed as a potential conflict of interest.
